# Warfarin-induced Eosinophilia in a Child with urkitt Lymphoma: A Case Report

**Published:** 2015

**Authors:** Kourosh Goudarzipour, Farid Ghazizadeh, Hesameddin Hoseini Tavassol, Behdad Behnam

**Affiliations:** a *Pediatric Congenital Hematologic Disorders Research Center of Shahid Beheshti University of Medical Sciences, Tehran, Iran.*; b *Functional Neurosurgery Research Center, Shohada Tajrish Hospital, Shahid Beheshti University of Medical Sciences, Tehran, Iran.*

**Keywords:** Warfarin, Eosinophilia, Anticoagulant therapy, Burkitt lymphoma, Pediatrics

## Abstract

An important complication of chemotherapy is thromboembolic events that can occur during treatment course. In this way, Warfarin can be used as an efficient prophylactic agent to prevent these complications. Although bleeding is a common adverse effect of Warfarin, eosinophilia is a rare side effect of this drug. We have reported a 5-year-old boy with Burkitt lymphoma who underwent chemotherapy. In the course of chemotherapy, because of thrombosis of the left jugular vein, we initiated Warfarin as a prophylactic drug for TEE secondary to chemotherapy. Following Warfarin initiation, eosinophilia appeared and subsequent to cessation of drug, eosinophilia disappeared.

This case is presented to point out physicians to consider eosinophilia as a rare adverse-effect of Warfarin and monitor blood cell differentiation regularly during the course of treatment with this drug.

## Introduction

In normal circumstances, eosinophils just account for about 1–3% of human peripheral blood leukocytes. (1) The term "eosinophilia" defined when eosinophil count rises up to 1,500/μL. Eosinophilia is classified into three parts: mild (400-1,500/μL), moderate (1,500-5,000/μL), and high (>5,000/μL) levels (2).

 Eosinophilia occurs in different conditions including allergies, parasitic infections, hematologic and oncologic disorders, *etc*. Orderly, helminthic infections and atopic disorders are the most common causes of eosinophilia worldwide and in developed countries.

In order to diagnose the etiology of eosinophilia, precise patient’s history is needed. It might include atopic signs such as wheezing or eczema; history of traveling to endemic areas of different parasitic infections; close contact to pets; symptoms those can reveal cancers; drug history that might indicate hypersensitivity reaction; *etc*. (1). 

Furthermore, eosinophilia has been reported as a rare side-effect following treatment with Warfarin (3, 4).

An important complication of chemotherapy is thromboembolic events that can occur during treatment course. In this way, Warfarin can be used as an efficient prophylactic agent to prevent these complications (5). Although eosinophilia is one of the rare side effects of this drug, it was seen in our case.

In this article, we have reported a rare case of Warfarin-induced eosinophilia. In this 5-year-old boy, we initiated Warfarin which led to presentation of eosinophilia following Warfarin prescription. To our best knowledge, this is the first pediatric case of eosinophilia associated with Warfarin therapy.

## Experimental

*Case report*

We have reported a 5-year-old boy who had been referred to our hospital with a history of cervical mass. He had been complaining of the left side cervical mass which was the mandibular lymphadenopathy. Consequently, excisional biopsy was performed and revealed the evidences of Burkitt lymphoma. Further evaluations were carried out and he diagnosed with Burkitt lymphoma, stage 4, with initial bone marrow involvement afterwards. The primary site of tumor was a mass in left side of neck and bone marrow involvement. Therefore, the patient underwent chemotherapy. He was being treated according to “treatment protocol for B-cell ALL and B-cell NHL” which includes Cyclophosamide, Vincristine, Methotrexate, Prednisone, Hydrocortisone, *etc*.) and four doses of Rituximab (6). Patient responded well to the treatment, bone marrow involvement recovered and only a residual mass in the primary site of the tumor remained. In this way second-look was done and the residual mass was resected entirely. In pathologic evaluation, no viable tumoral cells were seen in the resected mass and included only necrotic tissue.

After the second-look the chemotherapy was continued. In the course of chemotherapy the patient had been complaining of frequent pain in his neck. Consequently, he underwent cervical CT-Scan and it demonstrated a calcified soft tissue mass in the retro mandibular area of left side of the neck with thrombosis of left jugular vein. The CT-Scan findings was confirmed by Doppler ultra-sonography as a heterogeneous and hypo-echo lesion with 28*10 mm size that located in the left jugular vein from mandible angle extended to the base of neck which was in favor of thrombosis.

As a result, anticoagulant therapy was initiated with Enoxaparin, 15 mg (1 mg\Kg) twice a day. In the 5^th^ day of Enoxaparin prescription, Warfarin 15 mg (1 mg\Kg) was initiated and INR ratio reached between 2 to 3 afterwards. Subsequently, Enoxaparin was discontinued 8 days following warfarin initiation and warfarin dose was increased to 30 mg (2 mg\Kg) and continued (7). CBC of patient before starting Warfarin was normal but eosinophilia revealed two weeks after initiation of Warfarin in the serial CBC analysis. No sign and symptom was observed associated with eosinophilia.

There was no evidence of parasitic infections in the evaluation of eosinophilia. Stool examination showed no evidence of parasites or ova. Urine analysis and urine culture were normal and did not accompany with evidences of infections. The liver function indicators were totally normal. Bone marrow aspiration and biopsy were done and repeated after two weeks and all of them were normal those excluded hematologic and neoplastic etiologies of eosinophilia. Also, serum IgE level was normal that was against the allergic reaction.

There were no signs and symptoms of allergic disorders (*i.e*. atopic dermatitis, asthma, rhinitis, *etc*.). Furthermore, he had drug history of Ceftazidime, Vancomycin, Azithromycin and Cotrimoxazole in his antibacterial prophylactic drug regimen.

All of our blood samples were taken in the morning those showed a steady increase in the amount of eosinophils. The value of PT-INR ranged from 1.10 to 2.25 under Warfarin administration. No relation was seen between the PT-INR and eosinophil count. The eosinophil count increased up to 32% with WBC count of 4900/μL (absolute eosinophil count of 1568/μL) after two weeks Warfarin therapy, and after 35 days of Warfarin therapy the eosinophil fraction reached above 65% with WBC count of 5800/μL (absolute eosinophil count of 3770/μL). At first, all of his drugs, except Warfarin, were discontinued; however, the eosinophil count did not decrease following cessation. In the second step, Warfarin was terminated and Enoxaparin was initiated again instead of Warfarin for anticoagulation therapy. Consequently, the eosinophil level fell within a week and during a period of one month it returned to its normal range. As a result, it appeared that Warfarin was responsible for the rise of eosinophil numbers. However Warfarin was initiated again for the second time in the process of treatment in the third step, which caused an increase in eosinophil count up to 23% with WBC count of 5600/μL (absolute eosinophil count of 1288/μL) and rapid drug discontinuation. The patient was not rechallenged for the third time with Warfarin and received only Enoxaparin for 2.5 months and thrombosis was resolved.

## Discussion

Eosinophilia can be associated with different conditions such as: Allergic disorders, parasitic infection, eosinophilic enteritis, pneumonitis and idiopathic hypereosinophilic syndrome. Warfarin-induced eosinophilia is a rare etiology among them, though it was seen in our case. Few evidences have reported eosinophilia during the course of Warfarin therapy. 

The first report of Warfarin-induced eosinophilia has been reported by Hall and Link. In their case eighteen days after administration of Warfarin, laboratory findings show leukocytosis (19,000/μL) with eosinophilia of 51% (11,046/μL). The drug was stopped and twelve days following to cessation, eosinophil count decreased to 10.9% (676/μL) (8).

In 1995, Taishi Kuwahara *et al.* published a paper in which they described a case with Warfarin-induced eosinophilia. This case was a 51-year-old man. His symptoms were dry cough and low grade fever. Warfarin had been administrated subsequent to coronary artery bypass graft. CBC showed a WBC count of 10,600/μL with 18.1% eosinophils (1,919/μL), nine months following prescription of the Warfarin. Subsequent to discontinuation of Warfarin the eosinophil count decreased below 8% in two weeks. Regardless to this sequence, Warfarin was initiated again and eosinophils increased again up to 17% a week later. Therefore Warfarin was stopped again and in ten days eosinophil count decreased to its normal range. There were no other etiologies for eosinophilia in further evaluations (3).

In 2012, Masakazu Teragaki and et al. reported a case of Warfarin induced eosinophilia. This report is about a man who had an aortic valve replacement and following to surgery Warfarin was initiated. After that, eosinophilia was seen. However, subsequent to cessation of Warfarin, eosinophil count returned to its normal range again and it appeared that Warfarin was the cause of increase in eosinophil count (4).

In our case, the eosinophil fraction reached over 32% after two weeks Warfarin therapy with WBC: 4900/μL, Hb: 11 g/dl, mono: 4%, PMN: 50%, lymph: 12%, baso: 2%. The differential cell count reached above 65% of eosinophils on the 35th day of Warfarin therapy. The eosinophil count reduced and it returned to the normal range in one month after cessation of Warfarin. The eosinophil fraction increased up to 23% following to second prescription of Warfarin. This sequence of events, suggests that Warfarin is the cause of eosinophilia in this patient.

To our best knowledge, this is the first report of Warfarin-induced eosinophilia in pediatric patients.

## Results

Today, we have to be aware of adverse effects of Warfarin that is used more than before with recent progressions in care and treatment of pediatric diseases. It seems reasonable to regularly monitor blood cell differentiation during treatment with Warfarin and medical personnel should be aware of the potential of this complication.
